# Postoperative non‐steroidal anti‐inflammatory drugs and anastomotic leakage after gastrointestinal anastomoses: Systematic review and meta‐analysis

**DOI:** 10.1002/ags3.12300

**Published:** 2019-12-02

**Authors:** Supaschin Jamjittrong, Akihisa Matsuda, Satoshi Matsumoto, Tunyaporn Kamonvarapitak, Nobuyuki Sakurazawa, Youichi Kawano, Takeshi Yamada, Hideyuki Suzuki, Masao Miyashita, Hiroshi Yoshida

**Affiliations:** ^1^ Department of Surgery Nippon Medical School Chiba Hokusoh Hospital Chiba Japan; ^2^ Department of Surgery Queen Savang Vadhana Memorial Hospital Sri Racha Thailand; ^3^ Department of Gastrointestinal Hepato‐Biliary‐Pancreatic Surgery Nippon Medical School Tokyo Japan

**Keywords:** anastomotic leakage, cyclooxygenase inhibitor, gastrointestinal surgery, meta‐analysis, non‐steroidal anti‐inflammatory drugs

## Abstract

**Aim:**

Non‐steroidal anti‐inflammatory drugs (NSAIDs) are commonly used to control postoperative pain; however, their postoperative use has been associated with anastomotic leakage after gastrointestinal surgery. This systematic review and meta‐analysis aimed to determine the correlation between the use of NSAIDs and anastomotic leakage.

**Methods:**

We conducted a comprehensive electronic literature search up to August 2018 to identify studies comparing anastomotic leakage in patients with and without postoperative NSAID use following gastrointestinal surgery. We then carried out a meta‐analysis using random‐effects models to calculate odds ratios (OR) with 95% confidence intervals (CI).

**Results:**

Twenty‐four studies were included in this meta‐analysis, including a total of 31 877 patients. Meta‐analysis showed a significant association between NSAID use and anastomotic leakage (OR 1.73; 95% CI = 1.31‐2.29, *P* < .0001). Subgroup analyses showed that non‐selective NSAIDs, but not selective cyclooxygenase‐2 inhibitors, were significantly associated with anastomotic leakage. However there was no significant subgroup difference between selective and non‐selective NSAIDs.

**Conclusion:**

Results of this meta‐analysis indicate that postoperative NSAID use is associated with anastomotic leakage following gastrointestinal surgeries. Caution is warranted when using NSAIDs for postoperative analgesic control in patients with gastrointestinal anastomoses.

## Graphical abstract

The results of this meta‐analysis indicate that postoperative NSAID use is associated with anastomotic leakage following gastrointestinal surgeries. Although the difference was not significant, selective NSAIDs may be safer than non‐selective NSAIDs.

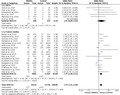

## 1 INTRODUCTION

Anastomotic leakage has long been a concern among gastrointestinal surgeons. Its occurrence not only causes postoperative morbidity and mortality, but also lengthens hospital stay and increases hospital costs.^1^, ^2^ Importantly, anastomotic leakage worsens oncological outcomes in patients with resectable and curable malignancies, leading to poorer disease‐free survival, overall survival, and functional outcome.^3^, ^4^

Multiple factors contribute to anastomotic leakage, and its incidence varies depending on the location of the anastomosis. Esophageal anastomoses have the highest incidence of leakage, and gastric anastomoses the lowest incidence, whereas the incidence of colorectal anastomotic leakage differs among publications and anastomosis sites, ranging from 1% to 20%.^5^

The early recovery after surgery protocol has been proposed to reduce postoperative stress. The protocol aims to promote postoperative recovery, reduce hospital stay and, most importantly, reduce postoperative complications, especially cardiovascular and pulmonary complications.^6^ Non‐steroidal anti‐inflammatory drugs (NSAIDs) play a major part in this protocol as a means of postoperative pain control. However, application of the early recovery after surgery protocol has been associated with an increased incidence of anastomotic leakage,^7^ and it has been suggested that NSAIDs may be a causative factor in impaired anastomotic healing.

Many potential mechanisms have been proposed to explain how postoperative NSAID use may cause anastomotic leakage. NSAIDs decreased protective prostaglandins, and inhibited mucosal cyclooxygenase (COX)‐1, intestinal epithelial cell migration, and mucosal restitution in animal models^8^ which, in turn, reduced anastomotic tensile strength and collagen deposition causing delayed anastomotic healing.^9^, ^10^, ^11^

Previous reviews have examined the correlation between postoperative NSAID use and anastomotic leakage, but most have considered colorectal anastomoses only.^7^, ^12^ However, we suggest that the mechanisms shown in animal models may be applicable to all gastrointestinal anastomoses. Furthermore, it is also possible that selective COX‐2 inhibitors may be safer than non‐selective NSAIDs in terms of preventing anastomotic leakage based on the above‐mentioned mechanism.

The primary objective of this systematic review and meta‐analysis was to determine the effect of postoperative NSAID use on gastrointestinal anastomotic leakage, regardless of the site of anastomosis. The secondary objective was to compare the anastomotic leakage risk between non‐selective NSAIDs and selective COX‐2 inhibitors.

## 2 METHODS

### 2.1 Search strategy

We conducted a literature search of the Medline, PubMed, Cochrane Library, http://clinicaltrial.gov, and Web of Science databases up to August 2018. The search was limited to English language and human studies. The search terms used were “Anastomosis or anastomotic leakage” AND “NSAIDs” [MesH term]. Additional articles were retrieved by manually searching the reference lists of the included studies and other reviews.

### 2.2 Selection criteria

Studies were included if they met the following criteria: (i) study with anastomosis of the gastrointestinal tract; (ii) study compared postoperative NSAID use with non‐use; and (iii) investigations reported anastomotic leakage. Case reports or reports with incomplete data were excluded.

### 2.3 Data extraction

The studies were independently and critically assessed by two authors using a standard protocol and discrepancies were resolved by consensus. Extracted data included study design, number of institutes, definition of anastomotic leakage, operative diagnosis, location of anastomosis, urgency of surgery, type of NSAIDs, sample size, and numbers of anastomotic leakage per group.

### 2.4 Quality assessment

Qualities of the included studies were assessed using the Jadad score^13^ and the Newcastle‐Ottawa scale (NOS)^14^ for randomized controlled trials (RCT) and observational studies, respectively. Studies were considered to be high quality if they had a Jadad score ≥3 or NOS ≥7.

### 2.5 Data synthesis and meta‐analysis

Meta‐analysis was done by computing the OR from the original data using the Cochrane‐Mantel‐Haenszel method, with 95% CI. *P* ≤ .05 was considered significant in all analyses. Data analysis was carried out using Review Manager (RevMan) v5.3 software (Cochrane Collaboration) and a random‐effect model was used for graphical presentation. Statistical heterogeneity was quantified using I^2^ statistics and Cochrane Q tests. I^2^ values >50% indicated heterogeneity.^15^ In the presence of heterogeneity, we conducted subgroup and meta‐regression analyses to determine if the inter‐study variation could be explained by certain co‐variates, including type of study, NSAID class, NSAID administration, urgency of surgery, location of anastomosis, and operative diagnosis. Sensitivity analyses were done to assess the impact of individual potential confounding variables. Publication bias was assessed visually by funnel plot, and asymmetry was assessed formally by rank correlation test (Begg’s test).^16^ Publication bias was analyzed using WINPEPI software.^17^

## 3 RESULTS

### 3.1 Study selection

**Figure 1:**
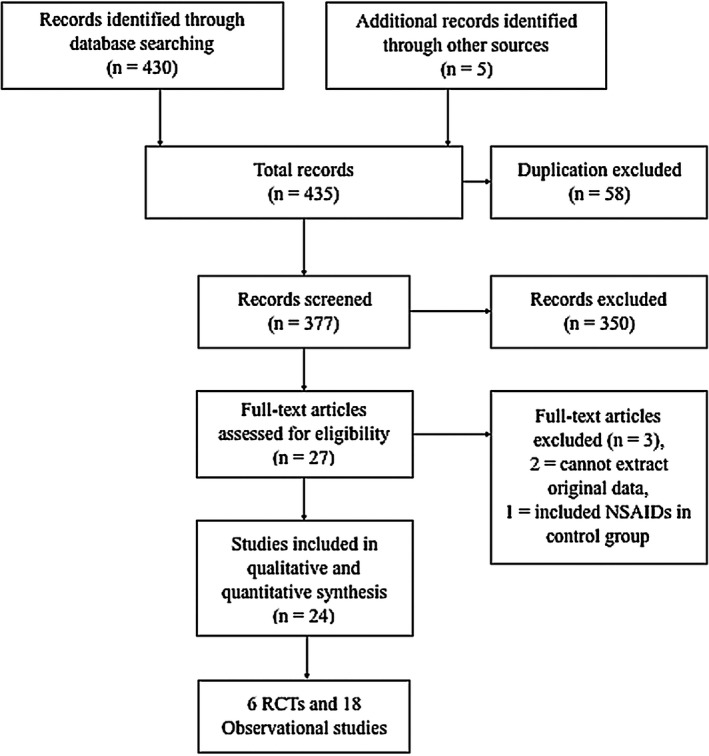
PRISMA flow shows study selection process. NSAIDs, non‐steroidal anti‐inflammatory drugs; RCT, randomized controlled trial

The initial systematic search identified 430 studies and an additional search for reviews identified a further five studies. After adjusting for duplicates and critical assessment, a total of six RCT^18^, ^19^, ^20^, ^21^, ^22^, ^23^ and 18 observational studies^24^, ^25^, ^26^, ^27^, ^28^, ^29^, ^30^, ^31^, ^32^, ^33^, ^34^, ^35^, ^36^, ^37^, ^38^, ^39^, ^40^, ^41^ were included in the meta‐analysis. The PRISMA flow diagram of the detailed literature search and selection process is shown in Figure 1. Of 27 full‐text article reviews, three were excluded from the quantitative analysis because we could not extract the original data from two, and the other study compared multimodal interventions in which NSAIDs were also distributed to the control group.

### 3.2 Characteristics of included studies

Six RCT and 18 observational studies were included in this meta‐analysis. Sample sizes varied from 40 to 220 for the RCT and from 75 to 13 082 for the observational studies. Most studies included the anastomotic location as colorectal anastomoses (four RCT,^18^, ^19^, ^21^, ^22^ 13 observational studies^24^, ^25^, ^26^, ^27^, ^28^, ^29^, ^31^, ^32^, ^34^, ^35^, ^36^, ^38^, ^40^), a diagnosis of malignancy (three RCT,^19^, ^20^, ^21^ six observational studies^24^, ^28^, ^36^, ^38^, ^40^, ^41^), and surgery carried out as an elective procedure (all RCT, 12 observational studies^24^, ^25^, ^26^, ^28^, ^29^, ^31^, ^32^, ^35^, ^36^, ^38^, ^40^, ^41^). Most studies reported the classes of NSAIDs used, except for five observational studies, from some of which we were able to extract the original data. Data on non‐selective NSAIDs were extracted from 15 studies^18^, ^19^, ^21^, ^22^, ^23^, ^24^, ^26^, ^27^, ^28^, ^31^, ^36^, ^37^, ^38^, ^40^, ^41^ and on selective COX‐2 inhibitors from eight studies.^20^, ^23^, ^25^, ^27^, ^29^, ^35^, ^38^, ^40^ Quality assessment showed that all the RCT and all but two of the observational studies were high quality,^24^, ^35^ with the two observational studies considered low quality. Characteristics of the included studies are outlined in Table 1.

**Table 1:** Characteristics of included studies to determine the correlation between the use of NSAIDs and anastomotic leakage

Author, year	Study design	Country, Institute	Recruitment period	Definition of AL	Diagnosis	Location of anastomosis	Urgency of surgery	N	NSAIDs administration	Quality assessment^a^
Chen,^18^ 2005	RCT, Double‐blind	Taiwan, single	2003	NR	Mixed	Colorectal	Elective	74	PCA: ketolorac 1.2 g/mL + morphine 1 mg/mL 2 mL bolus and 10 min lockout until pain score <3	5
Schlachta,^19^ 2007	RCT, Double‐blind	Canada, single	2002‐2005	NR	Mixed (Cancer 50%)	Colorectal	Elective	44	Ketolorac 30 mg IV every 6 h for 2 d after operation	3
Sim,^20^ 2007	RCT, Double‐blind	Singapore, single	2002‐2004	NR	Mixed (Cancer 94.9%)	Mixed (Colorectal 94.9%)	Elective	79	Valdecoxib 40 mg orally once pre‐operation and once daily for 5 d after operation	2
Xu,^21^ 2008	RCT, Double‐blind	China, single	2006‐2007	NR	Cancer	Colorectal	Elective	40	Flurbiprofen 1 mg/kg IV 30 min before and 6 h after skin incision	5
Chen,^22^ 2009	RCT, Double‐blind	Taiwan, single	2006‐2007	NR	Mixed	Colorectal	Elective	102	PCA: ketolorac 1.2 g/mL + morphine 1 mg/mL 2 mL bolus and 10 min lockout until pain score <3	4
Wattchow,^23^ 2009	RCT, Double‐blind	Australia, 2 institutes	2003‐2006	NR	Mixed	Mixed (Colorectal 99%, Small intestine 1%)	Elective	220	Celecoxib 100 mg or Diclofenac 50 mg orally twice daily for 7 d or until discharge	4
Rosenberg,^24^ 2007	Retrospective cohort	Denmark, Single	2004‐2006	NR	Cancer	Colorectal	Elective	310	Diclofenac 75 mg twice daily, Not reported duration	5
Klein,^26^ 2009	Retrospective case‐control	Denmark, Single	2004‐2007	Leak requiring reoperation	Mixed (Cancer 96%)	Colorectal	Elective	75	Diclofenac 150 mg/d, Not reported duration	7
Holte,^25^ 2009	Retrospective cohort	Denmark, Single	1997‐2006	Radiologic finding or intra‐operative finding or clinical finding	NR	Colon	Elective	502	Ibuprofen 600 mg every 8 h or Celecoxib 200 mg every 12 h at POD 2‐8	7
Gorissen,^27^ 2012	Retrospective cohort	Netherlands, 2 institutes	2008‐2010	Radiologic finding or intra‐operative finding or clinical finding	Mixed (Cancer 72%)	Colorectal	Mixed (Elective 86.4%)	795	NSAIDs use within POD 5	8
Klein,^28^ 2012	Retrospective cohort	Denmark, 6 institutes	2006‐2009	Leak requring reoperation	Cancer	Colorectal	Elective	2752	NSAIDs use at least 2 d within POD 7	9
Zittel,^29^ 2013	Retrospective cohort	Sweden, single	2008‐2009	NR	Mixed (Cancer 57.6%)	Colorectal	Elective	205	Etoricoxib 120 mg once daily, Not reported duration	8
Subendran,^32^ 2014	Retrospective case‐control	Canada, single	2001‐2012	Radiologic finding or intra‐operative finding	Mixed (IBD 65.6%, cancer 34.4%)	Colorectal	Elective	262	NSAIDs use within POD 5	8
Saleh,^31^ 2014	Retrospective cohort	Canada, single	2004‐2011	Document at reoperation or Radiological finding	Mixed (Cancer 65.5%)	Colorectal	Elective	731	NSAIDs use within POD 5	8
STARSurg UK,^30^ 2014	Prospective cohort	UK, multi‐institutes	2013	Radiologic finding or intra‐operative finding or clinical finding	Mixed (Cancer 62.1%)	Mixed (Colorectal 75.9%)	Mixed (Elective 72.1%)	1503	NSAIDs use within POD 2	8
Paulsir,^34^ 2015	Retrospective cohort	USA, multi‐institutes	2012‐2014	Leaks requiring antibiotic or intervention or reoperation	NR	Colorectal	Mixed (Elective 78.6%)	4360	NSAIDs use within POD 1	9
Hakkarainen,^33^ 2015	Retrospective cohort	USA, 47 institutes	2006‐2010	Leak requiring percutaneous drainage or reoperaion	NR	Bariatic, Colorectal	Mixed (Elective 87.6%)	13082	NSAIDs use within POD 1	9
Raju,^35^ 2015	Retrospective cohort	Australia, 2 institutes	2008‐2014	Leak requiring percutaneous drainage or reoperaion	Mixed (Cancer 70.6%)	Colorectal	Elective	267	Celecoxib 100 mg twice daily start at 2 h before operation to POD 7	6
Bakker,^36^ 2016	Retrospective cohort	Netherlands, single	2006‐2013	Leak requiring percutaneous drainage or reoperaion	Cancer	Colorectal	Elective	856	NSAIDs use at least 2 d until discharge	8
Rutegard,^38^ 2016	Retrospective cohort	Sweden, multi‐institutes	2007‐2012	Leak requiring percutaneous drainage or reoperaion	Cancer	Rectum	Elective	2605	NSAIDs use within POD 10	8
Rushfeldt,^37^ 2016	Retrospective cohort with propensity score analysis	Norway, Single	2007‐2009	NR	Mixed (Cancer 52.8%)	Mixed (Colorectal 73.4%)	Mixed (Elective 88%)	428	NSAIDs use within POD 5	8
Haddad,^39^ 2017	Retrospective cohort	USA, multi‐institutes	2013‐2015	NR	Trauma	Mixed (Small intestine 93.4%, Colorectal 6.6%)	Emergency	533	NSAIDs use 7 d prior to operation up to POD 14	7
Fjederholt,^41^ 2018	Retrospective cohort	Denmark, 2 institutes	2003‐2012	Radiologic finding or endoscopic finding	Cancer	Esophagojejunostomy	Elective	556	NSAIDs use within POD 7	9
Hultberg,^40^ 2017	Retrospective cohort	Sweden, 15 institutes	2007‐2013	Radiologic finding or intra‐operative finding or clinical finding or Endoscopic finding	Cancer	Rectal	Elective	1495	NSAIDs use at least 2 d within POD 7	9

Abbreviations: AL, anastomotic leakage; IBD, inflammatory bowel disease; NR, not reported; NSAIDs, non‐steroidal anti‐inflammatory drugs; PCA, patient controlled analgesia; POD, postoperative day; RCT, randomised controlled trial.

a Quality assessment for RCT and observational studies using Jadad score and Newcastle‐Ottawa scale (NOS) for randomised controlled trials (RCTs) and observational studies, respectively.

### 3.3 Association of NSAIDs with anastomotic leakage

**Figure 2:**
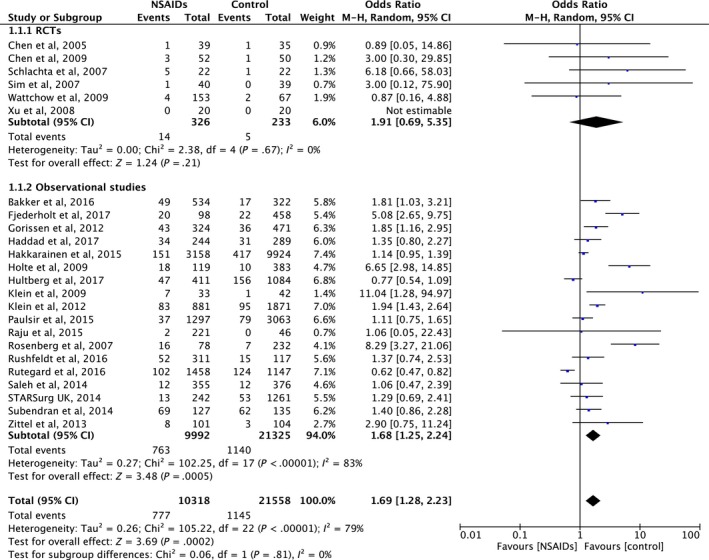
Forrest plot of meta‐analysis between randomized controlled trials (RCT) and observational studies. NSAIDs, non‐steroidal anti‐inflammatory drugs

**Figure 3:**
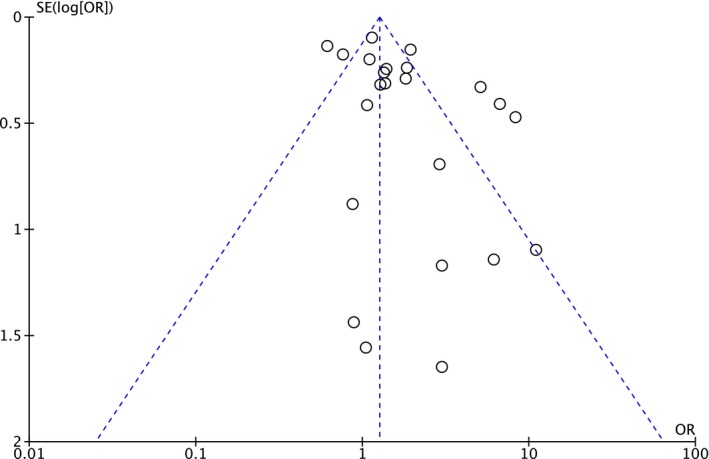
Funnel plot with pseudo 95% CI (random‐effect model). OR, odds ratio; SE, study effect

Overall anastomotic leakage rate in this study was 6.0% (1922/31 877). Patients who received NSAIDs postoperatively had a higher leakage rate (7.5%; 777/10 318) than those without NSAIDs (5.3%; 1145/21 558). Meta‐analysis showed a significantly higher rate of anastomotic leakage after postoperative NSAID use (pooled OR 1.73, 95% CI 1.31‐2.29, *P* < .001), but with evidence of heterogeneity across the included studies (I^2^ = 80%, Cochrane Q test *P* < .00001) (Figure 2). The funnel plot appeared relatively symmetrical, suggesting no publication bias, as confirmed by Begg’s test (*P* = .444) (Figure 3). There was some discrepancy in the results between the study types: RCT showed a non‐significant difference in anastomotic leakage between the NSAID and placebo groups (pooled OR 1.91, 95%CI 0.69‐5.35, *P* = .67) without heterogeneity (I^2^ = 0%, Cochrane Q test *P* = .67), whereas observational studies found a significantly higher leakage rate after postoperative NSAID use (OR 1.72, 95%CI 1.28‐2.31, *P* < .001) with evidence of heterogeneity (I^2^ = 84%, Cochrane Q test *P* < .001) (Figure 2).

### 3.4 Protocol‐based versus non‐systematic NSAIDs use

**Figure 4:**
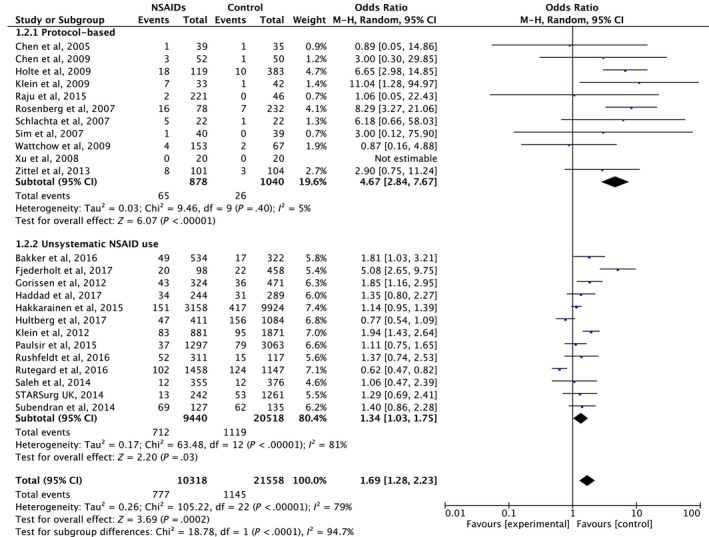
Forrest plot of meta‐analysis between protocol‐based non‐steroidal anti‐inflammatory drugs (NSAIDs) use and non‐systematic NSAIDs use

To investigate the effect of NSAID dose on anastomotic leakage, we categorized NSAID use in the included studies into protocol‐based and non‐systematic use. In the protocol‐based group, NSAIDs were given according to the institutional protocol (11 studies; n = 1918), whereas in the non‐systematic group, NSAIDs were given at any given time during the postoperative period (13 studies; n = 30 140). Details of NSAID use are shown in Table 1. The protocol‐based group had a significantly higher anastomotic leakage rate compared with non‐users (pooled OR 4.67, 95% CI 2.84‐7.67, *P* < .001) without evidence of heterogeneity (I^2^ = 5%, Cochrane Q test *P* = .40), whereas the non‐systematic group also had a significantly increased risk for anastomotic leakage compared with non‐users (pooled OR 1.38, 95% CI 1.06‐0.181, *P* = .02), but with evidence of heterogeneity (I^2^ = 82%, Cochrane Q test *P* < .001). However, there was a statistically significant subgroup difference between the protocol‐based group and the non‐systematic group (*P* < .001) (Figure 4).

### 3.5 Non‐selective NSAIDs versus selective COX‐2 inhibitors

**Figure 5:**
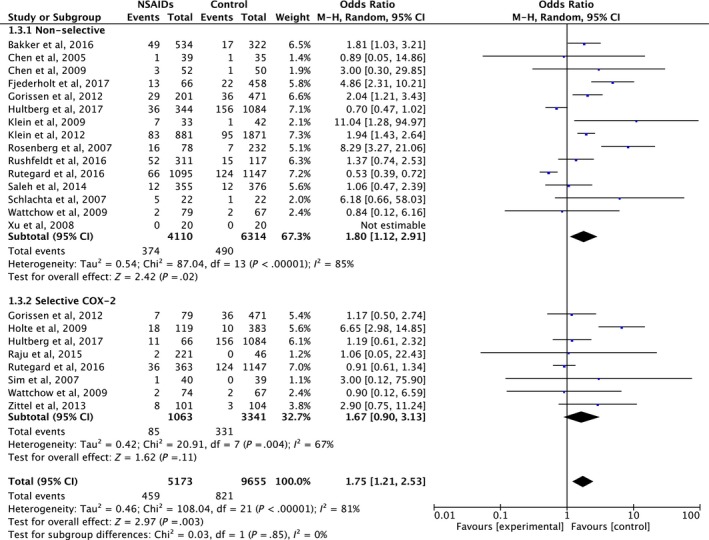
Forrest plot of meta‐analysis between non‐selective non‐steroidal anti‐inflammatory drugs (NSAIDs) and selective COX‐2 NSAIDs

Among all the included studies, we extracted information on non‐selective NSAID use from 15 (n = 4110) and on selective COX‐2 inhibitor use from eight (n = 1063) studies. Subgroup analysis showed that patients who received postoperative non‐selective NSAIDs had a significantly higher rate of anastomotic leakage than patients who did not receive NSAIDs (pooled OR 1.80, 95% CI 1.12‐2.91, *P* = .02) with evidence of heterogeneity (I^2^ = 85%, Cochrane Q test *P* < .00001). In contrast, the anastomotic leakage rate in patients taking selective COX‐2 inhibitors was not significantly higher than in those not taking NSAIDs (pooled OR = 1.67, 95% CI 0.90‐3.13, *P* = .11), with evidence of heterogeneity (I^2^ = 67%, Cochrane Q test *P* = .004). However, comparison between users of non‐selective and selective NSAIDs showed no significant subgroup difference (*P* = .85) (Figure 5).

### 3.6 Colorectal anastomoses versus other gastrointestinal anastomoses

We carried out subgroup analyses between studies restricted to colorectal anastomoses (17 studies; n = 15 475) and studies with anastomoses not limited to colorectal (seven studies; n = 16 538). Studies with colorectal anastomoses had significantly increased anastomotic leakage rates when perioperative NSAIDs were used (pooled OR 1.80, 95% CI 1.22‐2.66, *P* = .003), with evidence of heterogeneity (I^2^ = 83%, Cochrane Q test *P* < .00001). Studies of anastomoses of all sites also showed significantly higher rates of anastomotic leakage (pooled OR 1.61, 95% CI 1.25‐2.66, *P* = .02), with evidence of heterogeneity (I^2^ = 72%, Cochrane Q test *P* = .002). There were no subgroup differences between the two groups of studies (*P* = .85) (Figure S1).

### 3.7 Meta‐regression and sensitivity analyses

Meta‐regression analysis stratified by location of anastomoses showed pooled OR for anastomotic leakage of 1.80 (95% CI 1.22‐2.66, I^2^ = 83%) for colorectal anastomoses and 1.70 (95% CI 1.09‐2.66, I^2^ = 72%) for studies that were not limited to colorectal anastomoses. Meta‐regression analysis showed no significant difference between various anastomotic sites (*P* = .85). Furthermore, separate stratified and meta‐regression analyses showed no significant differences in the OR of anastomotic leakage rates after postoperative NSAID use in relation to the type of study, NSAID class, urgency of surgery, or operative diagnosis (Table 2).

**Table 2:** Stratified analysis and meta‐regression of included studies

	Studies	N	OR (95% CI)	*I* ^2^	Heterogeneity		
					χ^2^	*I* ^2^	*P* value
1. Type of studies							
RCTS	6	559	1.91 (0.69‐5.35)	0	0.06	0	.81
Cohort studies	18	31 317	1.68 (1.25‐2.24)	83			
2. NSAIDs class							
Non selective	15	10 424	1.80 (1.12‐2.91)	85	0.03	0	.85
Selective COX‐2	8	4404	1.67 (0.90‐3.13)	67			
3. Urgency of surgery							
Elective	18	11 175	2.08 (1.31‐3.29)	84	4.55	72	.03
Not limit to elective surgery	6	20 701	1.23 (1.06‐1.42)	0			
4. Location of anastamoses							
Colorectal	17	15 475	1.80 (1.22‐2.66)	83	0.20	0	.66
Not limit to colorectal	7	16 401	1.58 (1.04‐2.42)	72			
5. Diagnosis							
Cancer	7	8614	1.88 (0.96‐3.69)	93	0.31	0	.58
Not limit to cancer	17	23 262	1.54 (1.21‐1.96)	44			
6. NSAIDs administration							
Protocol based	11	1918	4.67 (2.84‐7.67)	5	18.78	94.7	<.0001
Unsystematic	13	29 958	1.34 (1.03‐1.75)	81			

Abbreviations: CI, confidence interval; NSAIDs, non‐steroidal anti‐inflammatory drugs; OR, odds ratio; RCT, randomized controlled trial

Sensitivity analyses were carried out to assess the impact of low‐quality studies (Table 1). Exclusion of the two low‐quality studies did not affect the significance of the results (pooled OR 1.61, 95% CI 1.22‐2.11, *P* < .001).

## 4 DISCUSSION

Numerous mechanisms have shown how NSAIDs can damage human intestines, although some remain controversial. Non‐selective NSAIDs have been associated with enterocyte mitochondrial dysfunction leading to increased epithelial permeability, invasion of luminal bacteria, neutrophil infiltration, and free radical production.^42^, ^43^, ^44^ Inhibition of COX by NSAIDs also decreases protective prostaglandins.^45^ Non‐selective NSAIDs and their acidic compounds can cause topical mucosal injury.^9^ However, most COX in the intestinal mucosal layer are COX‐1, and selective COX‐2 inhibitors may thus be more tolerable in the normal gastrointestinal tract.

Selective COX‐2 inhibitors and non‐selective NSAIDs confound the anastomotic healing process. Submucosal collagen fibers provide a core structure that determines tensile strength, and both selective COX‐2 inhibitors and non‐selective NSAIDs adversely affected this structure in an animal model which, in turn, led to decreased tensile strength of the anastomoses and reduced bursting pressure.^46^, ^47^, ^48^ NSAIDs also inhibited epithelial cell migration and mucosal restitution by depolarization and decreased surface expression of potassium channels.^8^ However, unlike in normal tissue, enterocytes express high levels of COX‐2 during inflammation, which catalyzes prostaglandin E2, resulting in increased vascular endothelial growth factor expression and angiogenesis.^49^

The above results and hypotheses shed doubt on the safety of postoperative NSAID use for analgesic control. Numerous previous meta‐analyses have shown significantly higher anastomotic leakage rates in patients given NSAIDs.^7^, ^12^, ^50^ The current systematic review and meta‐analysis confirmed the association between postoperative NSAID use and higher anastomotic leakage (pooled OR 1.73, 95% CI 1.31‐2.29, *P* < .001). However, our analysis of RCT did not show a significant effect of postoperative NSAIDs on anastomotic leakage rate compared with placebo. This meta‐analysis included only six RCT. Furthermore, the primary outcome of all RCT were not anastomotic leakage; therefore, we extracted corresponding data from each RCT. Finally, the sample size from RCT was very small compared to observational studies (n = 559 vs 31 499), which makes it relatively reasonable to integrate both study designs in order to make a conclusion from current evidence. From the result of no significant subgroup difference between studies, RCT and all designs, we believe that the controversial result may be explainable by the small sample sizes of the RCT, thus limiting their statistical power, rather than by the absence of a relationship between NSAIDs use and anastomotic leakage.

Subgroup analysis showed that patients taking NSAIDs according to hospital protocol had significantly higher rates of anastomotic leakage than those not taking NSAIDs (pooled OR 4.67, 95% CI 2.84‐7.67, *P* < .001), without evidence of heterogeneity (I^2^ = 5%, Cochrane Q test *P* = .40). Patients in the protocol‐based group were supposedly given NSAIDs in a regular way, with higher cumulative doses compared with the non‐systematic group. This suggests that the association between NSAID use and anastomotic leakage may be dose‐related, although further studies are needed to confirm this theory.

Subgroup analysis also showed that patients taking non‐selective NSAIDs had a significantly higher rate of anastomotic leakage than patients not taking NSAIDs (pooled OR 1.80, 95% CI 1.12‐2.91, *P* = .02). In contrast, selective COX‐2 inhibitors tended to increase the risk of anastomotic leakage, but the effect was not significant (pooled OR = 1.67, 95% CI 0.90‐3.13, *P* = .11). However, there was no significant subgroup difference between patients taking non‐selective and COX‐2‐selective NSAIDs. These results support the hypotheses that both classes of NSAIDs had adverse effects on anastomotic healing, leading to increased anastomotic leakage; however, non‐selective NSAIDs might cause greater damage then selective COX‐2 inhibitors by causing intestinal mucosal injury, at least in part.

In animal models, adverse effects of NSAIDs were found in both small intestine and colon resulting in increased anastomotic leakage rate.^8^, ^9^, ^11^, ^42^, ^44^ In human studies, consistent results were also reported regardless of anastomotic site; however, the majority were colorectal anastomoses. In our study, studies with colorectal anastomoses had significantly increased anastomotic leakage rates when perioperative NSAIDs were used (pooled OR 1.80, 95% CI 1.22‐2.66, *P* = .003). Consistently, studies of anastomoses of all sites also showed significantly higher rates of anastomotic leakage (pooled OR 1.61, 95% CI 1.25‐2.66, *P* = .02). There were no subgroup differences between the two groups of studies (*P* = .85). In fact, Fjederholt et al^41^ reported a strong association between NSAIDs use and the risk of anastomotic leakage (ketorolac; OR 6.05, 95% CI 2.71‐13.5) (other NSAIDs; OR 5.24, 95% CI 1.85‐14.8) after surgery for gastroesophageal junction only. Two other studies^33^, ^39^ of which majority of anastomosis site is not colorectal, were also included in our meta‐analysis. These results support our hypothesis that NSAIDs were associated with increased anastomotic leakage in all gastrointestinal anastomoses.

The present study had several limitations. First, our conclusions were mainly based on observational studies; however, subgroup analysis showed no significant subgroup difference between RCT and observational studies, suggesting that this potential bias was not significant. Second, there was statistical heterogeneity, and the included observational studies were clinically heterogenous in terms of patient characteristics, indications for surgery, and location of anastomoses. Although stratified and meta‐regression analyses showed no significant differences, heterogeneity decreased the validity of the results. Third, most of the included studies (17/24) only considered colorectal anastomoses, and the implication of the results for all gastrointestinal anastomoses might not be completely accurate.

In conclusion, postoperative NSAID use appears to be associated with an increased incidence of anastomotic leakage following gastrointestinal surgery. Selective COX‐2 inhibitors might be safer than non‐selective NSAIDs, although the results were inconclusive. Caution is warranted when using NSAIDs for postoperative analgesic control in patients with gastrointestinal anastomoses.

## DISCLOSURE

Conflicts of Interest: Authors declare no conflicts of interest for this article.
