# Supplementary figures and images for: Postoperative non‐steroidal anti‐inflammatory drugs and anastomotic leakage after gastrointestinal anastomoses: Systematic review and meta‐analysis
Source: Ann Gastroenterol Surg. 2019 Dec 2;4(1):64–75. doi: 10.1002/ags3.12300 (PMC6992684; doi:10.1002/ags3.12300)

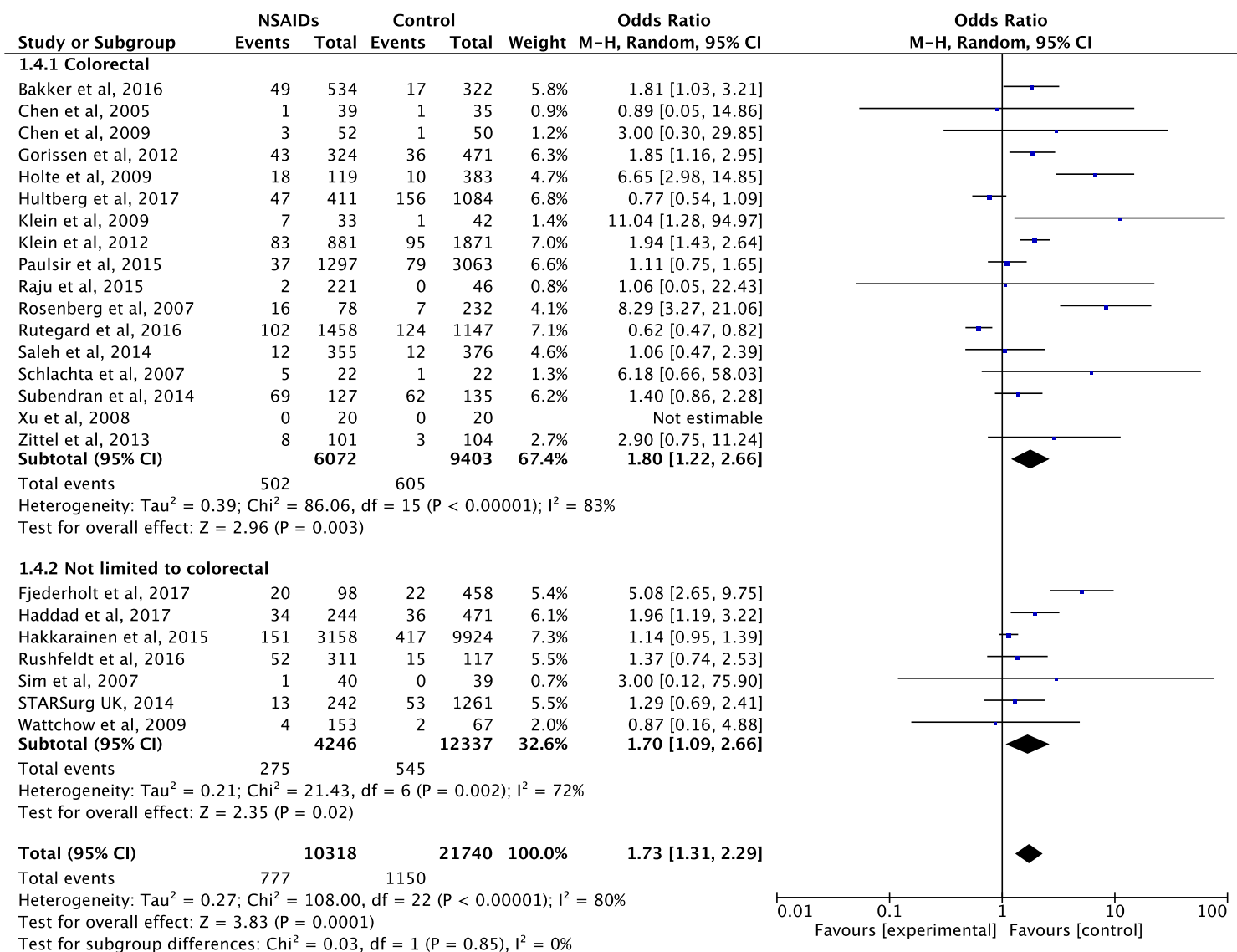

Supplement: Supplementary file 1 [file AGS3-4-64-s001.pdf]
